# Lentinan Protects against Nonalcoholic Fatty Liver Disease by Reducing Oxidative Stress and Apoptosis via the PPARα Pathway

**DOI:** 10.3390/metabo12010055

**Published:** 2022-01-10

**Authors:** Tingyi Du, Qin Fang, Zhihao Zhang, Chuanmeng Zhu, Renfan Xu, Guangzhi Chen, Yan Wang

**Affiliations:** 1 Division of Cardiology, Department of Internal Medicine, Tongji Hospital, Tongji Medical College, Huazhong University of Science and Technology, Wuhan 430030, China; dutingyiznh@163.com (T.D.); fangqin140716@126.com (Q.F.); zhihaotjh@163.com (Z.Z.); zhuchuanmengokok@163.com (C.Z.); 2 Department of Radiology, Zhongnan Hospital of Wuhan University, Wuhan 430071, China; 3 Department of Medical Ultrasound, Tongji Hospital, Tongji Medical College, Huazhong University of Science and Technology, Wuhan 430030, China; xurenfantjh@163.com

**Keywords:** lentinan, nonalcoholic fatty liver disease, PPARα, oxidative stress, apoptosis

## Abstract

Lentinan (LNT), a type of polysaccharide derived from *Lentinus edodes*, has manifested protective effects during liver injury and hepatocellular carcinoma, but little is known about its effects on nonalcoholic fatty liver disease (NAFLD). This study aimed to investigate whether LNT can affect the progression of NAFLD and the associated mechanisms. C57BL/6J mice were fed a normal chow diet or a high-fat diet (HFD) with or without LNT (6 mg/kg/d). AML12 cells were exposed to 200 μM palmitate acid (PA) with or without LNT (5 μg/mL). After 21 wk of the high-fat diet, LNT significantly decreased plasma triglyceride levels and liver lipid accumulation, reduced excessive reactive oxygen species production, and subsequently attenuated hepatic apoptosis in NAFLD mice. These effects were associated with increased PPARα levels, a decreased Bax/Bcl-2 ratio, and enhancement of the antioxidant defense system in vivo. Similar effects were also observed in cultured cells. More importantly, these protective effects of LNT on palmitate acid-treated AML12 cells were almost abolished by PPARα knockdown. In conclusion, this study demonstrates that LNT may ameliorate hepatic steatosis and decrease oxidative stress and apoptosis by activating the PPARα pathway and is a potential drug target for NAFLD.

## 1. Introduction

Nonalcoholic fatty liver disease (NAFLD) has become the most common chronic liver disease in the world [1,2]. In fact, approximately 25% of the global population is currently thought to have NAFLD [2,3]. NAFLD covers a spectrum of liver damage ranging from simple steatosis to more severe forms of liver injury, including nonalcoholic steatohepatitis (NASH), fibrosis, and hepatocellular carcinoma [4,5]. Furthermore, NAFLD is a strong risk factor for cardiovascular disease, atherosclerosis, Type 2 diabetes, and chronic kidney disease [3,6]. Currently, there are limited therapeutic options. Lifestyle modifications are difficult to achieve and sustain, and approved pharmacological therapy is lacking [7,8]. Rapid discovery of effective treatments for NAFLD is needed.

Whereas the underlying mechanism for the development and progression of NAFLD is complex and multifactorial. The “multiple-hit hypothesis” has been widely accepted for the development of NAFLD [9]. The “multiple-hit hypothesis” involves widespread metabolic dysfunction, including collaboration among genetic, metabolic and environmental factors, which promote the accumulation of fat in hepatocytes and successively cause inflammation, oxidative stress, apoptosis and fibrosis. However, fat accumulation in the liver caused by obesity and insulin resistance is still considered to be the hallmark feature determining NAFLD. The excess fat in the liver, especially free cholesterol and saturated fatty acids (SFAs), causes lipotoxicity and leads to organelle failure through mitochondrial dysfunction [10]. A dysfunctional mitochondrion can cause ROS formation by increasing the capacity to oxidize FA and causing oxidative stress due to an imbalance between the production of ROS and protective oxidants [11]. Oxidative stress can lead to hepatocellular damage through several mechanisms, including lipid peroxidation, which can directly activate the apoptotic Fas-ligand pathway and cell necrosis, and has even been implicated in causing fibrosis [12]. Apoptosis is also a key driver in terms of hepatocyte cell death in NASH [13]. Caspase3 cleavage and TUNEL tests were positive in liver tissue from both NASH patients and mouse models of NASH [14]. Noninvasive markers of apoptosis such as circulating CK18 levels are also increased in NASH patients and can predict the presence of NASH [15].

Lentinan (LNT) is a polysaccharide component extracted from *Lentinus edodes* that has a β-1,3-glucan structure [16] and has been found in many previous studies to have multiple functions, including immunomodulatory [17], antiviral [18], antitumor [19], antioxidative, and anti-inflammatory effects [20]. In clinical work, we observed that lentinan supplementation could significantly improve lipid levels in patients with hyperlipidemia. In addition, studies have demonstrated that *Lentinus edodes* intake could significantly decrease blood lipids in spontaneous hypertension rats [21]. Moreover, β-glucan extracted from other substances has also been shown to improve lipid metabolism-related diseases in numerous studies [22,23]. However, the protective effects of LNT on lipid metabolism in NAFLD and the associated molecular pathway have not yet been elucidated.

Peroxisome proliferator-activated receptor α (PPARα), a transcription factor of the NR1C family that is abundantly expressed in the liver [24], takes part in several aspects of lipid metabolism [25], including fatty acid degradation, synthesis, transport, storage, and lipoprotein metabolism [24]. PPARα knockout mice given a high-fat diet were found to have severe steatosis and hepatitis [26]. However, in wild-type mice, the PPARα agonist can improve the morphological changes in the liver by regulating intracellular lipid deposition, liver inflammation, oxidative stress, and fibrosis [27]. In addition, PPARα also suppressed antioxidative expression in high-fat diet (HFD)-induced NAFLD [28]. Moreover, the PPARα agonist fenofibrate significantly decreased apoptosis factors (Bax and Caspase3) in HFD-induced NAFLD [29].

In this study, we conducted both in vivo and in vitro experiments to explore the effect of LNT on NAFLD, focusing on its role in hepatic steatosis, oxidative stress, and apoptosis responses, and to elucidate its mechanism of action.

## 2. Results

### 2.1. LNT Prevented HFD-Induced Steatosis in Mice

**Figure 1:**
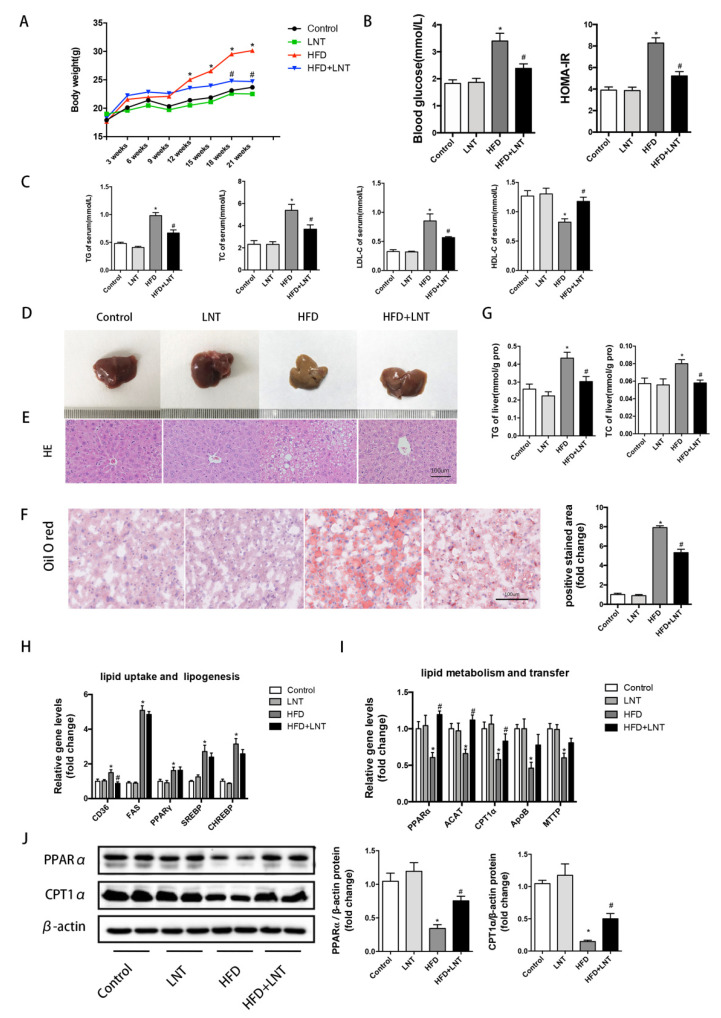
LNT ameliorated HFD-induced steatosis in mice. (**A**) Body weight, (**B**) blood glucose and HOMA-IR in four groups of mice. (**C**) Serum levels of TG, TC, LDL-C and HDL-C were determined. (**D**–**F**) Representative images of H&E and oil red O staining of the liver in mice. (**G**) Liver TG and TC content. (**H**,**I**) Hepatic mRNA levels of lipid uptake-, lipogenesis-, metabolism-, and transfer-related genes. (**J**) Representative Western blots and quantification of PPARα and CPT1α in the liver. Data are presented as means ± SEM (*n* = 8 per group). * *p* < 0.05 compared with the Control group. ^#^ *p* < 0.05 compared with the HFD group.

As mentioned in the flow chart of the animal experiment shown in Supplementary Materials Figure S1, LNT intervention persisted for 15 weeks. During Weeks 6 to 21, the HFD group exhibited rapid increases in body weight, while LNT significantly prevented the body weight gain induced by the high-fat diet (Figure 1A). Measured blood glucose and HOMA-IR results also showed a significant increase in the HFD group and a significant decrease in the LNT treatment group (Figure 1B). Serum TG, TC, and LDL-C, and liver TG and TC were significantly increased in the HFD group but were significantly suppressed by LNT (Figure 1C,G). In addition, LNT significantly increased the HDL-C levels in the serum (Figure 1C).

In the examination of liver tissues, livers exhibited an enlarged size and a faded color with a fat layer on the surface and a soft texture in the HFD group, while these changes were prevented by the LNT treatment (Figure 1D). In the HFD group, vacuolar degeneration inside the liver parenchyma cells was found by hematoxylin and eosin staining (Figure 1E) and a large accumulation of lipid droplets was found by oil red O staining (Figure 1F). However, the degree of steatosis was remarkably limited under the LNT treatment. These results demonstrated that LNT significantly prevented the progress of steatosis in NAFLD mice.

To further explore the effect of LNT on liver function injury induced by HFD, we measured the associated enzymes of liver function, serum AST and ALT levels. The results showed that LNT significantly decreased the increase in AST and ALT levels induced by HFD (Supplementary Materials Figure S3A). We also measured serum bile acid, ALP, and total bilirubin levels in various animal groups, and found that the high-fat diet increased serum bile acid, ALP, and total bilirubin levels. We noticed that LNT slightly decreased serum bile acid, ALP, and total bilirubin levels, but this did not reach statistical significance (Supplementary Materials Figure S3B). In addition, we analyzed the pro- and anti-inflammatory cytokines in both the serum and liver. LNT alleviated the increase in serum TNF-α and IL-6 levels in high-fat diet-treated mice; however, serum IL-10 levels did not significantly increase (Supplementary Materials Figure S4A). Moreover, we measured the levels of TNF-α, IL-6, and IL-10 mRNA and protein in liver tissues, which showed similar results with serum (Supplementary Materials Figure S4B,C). These results suggested that LNT prevented the increase in pro-inflammatory cytokines induced by HFD but had no significant effect on anti-inflammatory cytokines.

To investigate the mechanisms by which LNT prevented the progress of hepatic steatosis, the expression of genes associated with lipid uptake, lipogenesis, and lipid metabolism were detected in the liver. In the HFD group, the mRNA expression levels of CD36, FAS, PPARγ, SREBP, and CHREBP increased significantly, and PPARα, ACAT, CPT1α, ApoB, and MTTP decreased significantly compared with the Control group, while LNT treatment dramatically downregulated the expression of CD36 and upregulated the expression of PPARα, ACAT, and CPT1α compared with the HFD group (Figure 1H,I). The levels of PPARα and CPT1α, the key enzymes in lipid metabolism, were further determined in protein by Western blot analysis. The results also indicated that LNT significantly upregulated the PPARα and CPT1α protein levels compared with those in the HFD group (Figure 1J). In addition, we also detected the protein expression of AMPKα, p-AMPKα, and RXRα under the LNT treatment and found that LTN showed no detectable effects on these genes (Supplementary Materials Figure S5A,B). These data indicated that the PPARα may be involved in the effects of LNT on regulating steatosis in NAFLD mice.

### 2.2. LNT Protected against HFD-Induced Hepatic Oxidative Stress and Apoptosis in Mice

**Figure 2:**
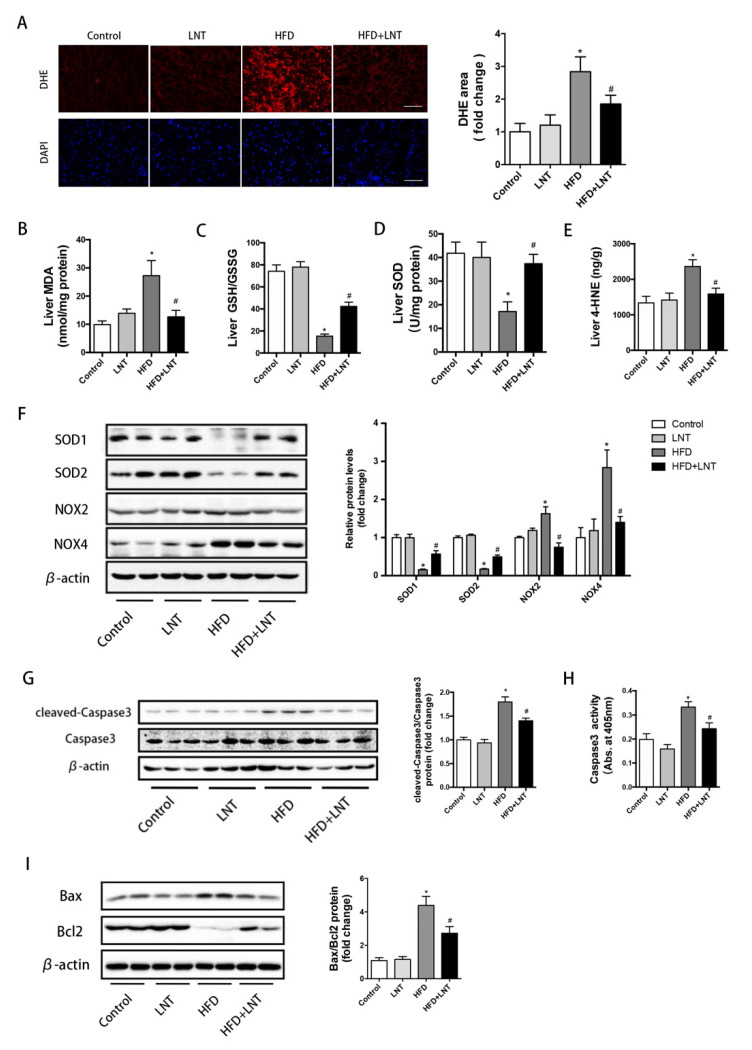
LNT suppressed HFD-induced oxidative stress and apoptosis in the liver. (**A**) Representative images and quantification of DHE staining of hepatic ROS production. (**B**–**E**) MDA, GSH/GSSH, SOD, and 4-HNE levels in the liver. (**F**) Representative Western blots and quantification of SOD1, SOD2, NOX2, and NOX4 in the liver. (**G**) Cleaved-Caspase3 expression by Western blot analysis. (**H**) Caspase3 activity in livers from the four treatment groups. (**I**) Representative Western blots and quantification for apoptosis-related proteins in the liver. Data are presented as the mean ± SEM (*n* = 8 per group). * *p* < 0.05 compared with the Control group. ^#^ *p* < 0.05 compared with the HFD group.

To evaluate the effects of LNT on oxidative stress in the hepatic tissue of NAFLD mice, we determined the levels of reactive oxygen species (ROS) and lipid peroxidation products, as well as the activity or content of enzymes that are known to be key regulators maintaining redox homeostasis. DHE staining indicated that ROS production in the liver was significantly elevated in the HFD group but was dramatically decreased under the LNT treatment (Figure 2A). In NAFLD mice, LNT significantly increased hepatic SOD and GSH/GSSG content, and significantly decreased hepatic MDA and 4-HNE, compared with the HFD group (Figure 2B–E). In addition, compared with the HFD group, hepatic antioxidant enzyme (SOD1, SOD2) protein levels were dramatically upregulated and hepatic pro-oxidant enzyme (NOX2, NOX4) protein levels were significantly downregulated under the LNT treatment (Figure 2F).

Compared with the HFD group, LNT significantly decreased cleaved-Caspase3 protein levels and Caspase3 activity (Figure 2G,H). Moreover, after the high-fat diet, the pro-apoptotic Bax protein was dramatically increased and the anti-apoptotic Bcl-2 protein was significantly decreased compared with the Control group, while these changes were prevented by LNT (Figure 2I). Taken together, all these results suggested that LNT significantly protected against hepatic oxidative stress and apoptosis in NAFLD mice.

### 2.3. LNT Prevented Lipid Accumulation in PA-Induced AML12 Cells

**Figure 3:**
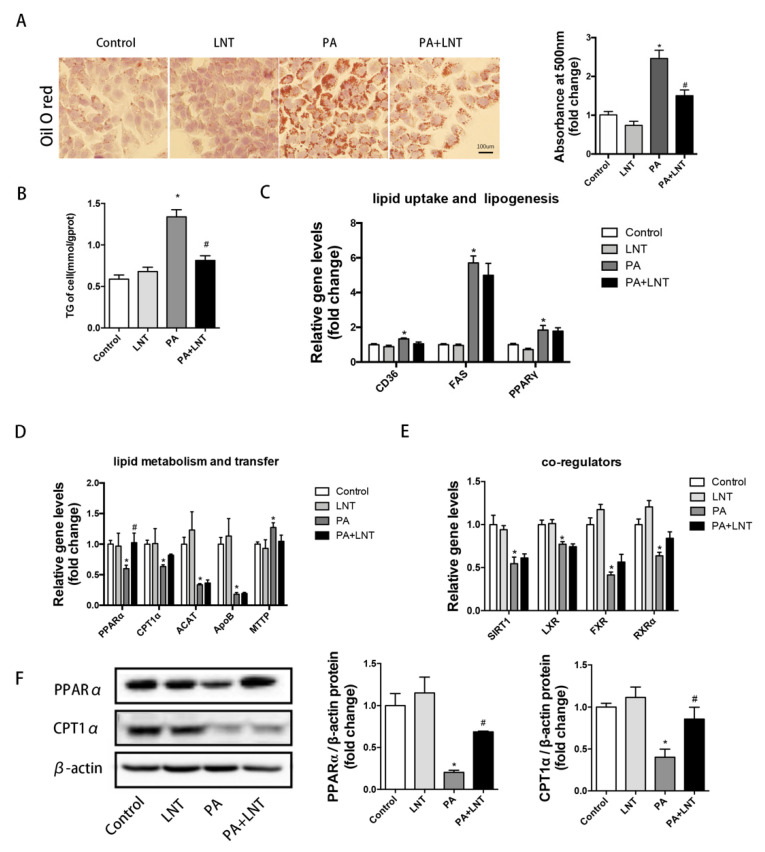
LNT alleviated lipid accumulation in PA-induced AML12 cells. (**A**,**B**) Oil red O staining and TG content in AML-12 cells. (**C**–**E**) mRNA levels of lipid uptake-, lipogenesis-, metabolism-, and transfer-related genes in AML-12 cells. (**F**) Representative Western blots and quantification of PPARα and CPT1α in AML-12 cells. Data are presented as the mean ± SEM (*n* = 3 per group). * *p* < 0.05 compared with the Control group. ^#^ *p* < 0.05 compared with the PA group.

To investigate the effects of LNT on PA-induced lipid deposition in vitro, we used AML-12 cells, which have been well documented as cellular models of NAFLD [30]. The cultured hepatocytes were treated with 5 μg/mL of LNT or palmitic acid for 24 h. Intracellular lipid content was assessed by oil red O staining and a triglyceride assay. Hepatocyte lipid droplets and triglyceride content were significantly increased in PA-induced AML12 cells. However, LNT dramatically prevented lipid deposition in AML12 cells (Figure 3A,B).

Consistent with the in vivo findings, LNT significantly decreased PPARα and CPT1α mRNA and protein levels in PA-treated AML-12 cells. However, LNT had no obvious effect on the expression of CD36, FAS, and PPAR-γ mRNA (Figure 3C,D,F). In addition, the co-regulators of PPARα genes, including SIRT1, LXR, FXR, and RXRα were significantly upregulated in PA-treated AML-12 cells, while LNT treatment had no significant effect (Figure 3E). Taken together, these findings indicated that LNT may prevent PA-induced lipid accumulation by activating the PPARα signaling pathway in vitro.

### 2.4. LNT Improved Oxidative Stress and Apoptosis in PA-Induced AML12 Cells

**Figure 4:**
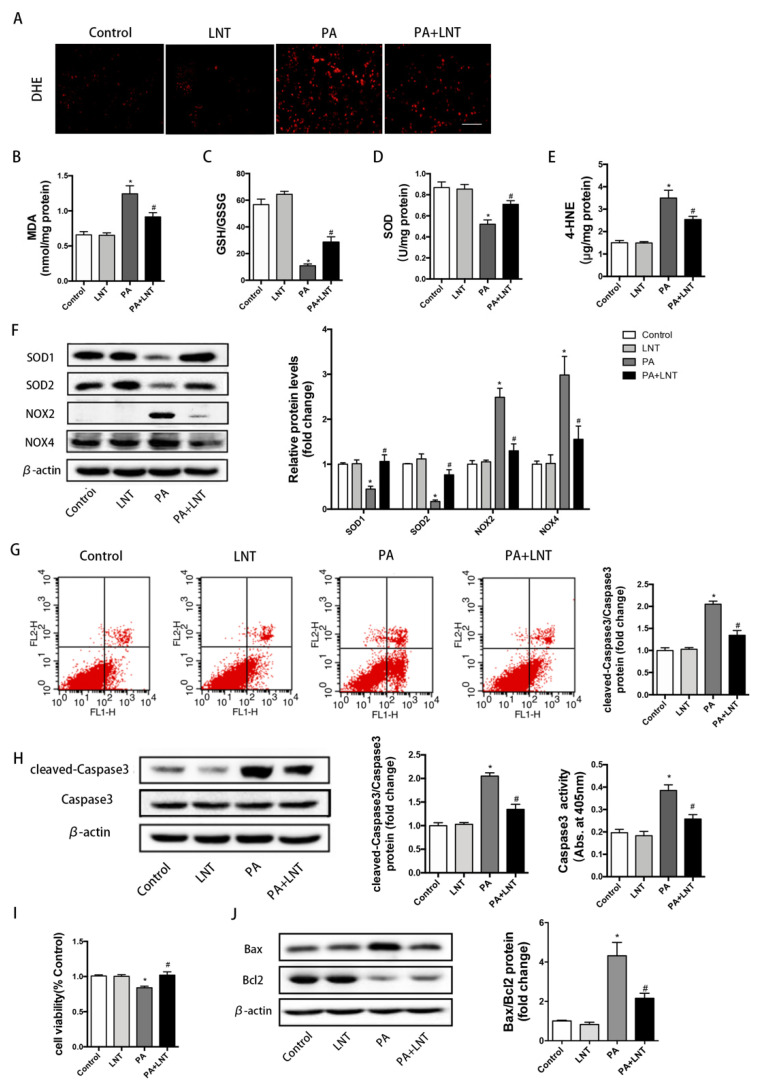
LNT improved oxidative stress and apoptosis in PA-induced AML12 cells. (**A**) DHE staining of AML-12 cells. (**B**–**E**) MDA, GSH/GSSH, SOD, and 4-HNE levels in AML-12 cells. (**F**) Representative Western blots and quantification of SOD1, SOD2, NOX2, and NOX4 in AML-12 cells. (**G**) Representative annexin-V-FITC/PI staining and quantification of apoptosis by flow cytometry. (**H**) Cleaved-Caspase3 expression by Western blot analysis and Caspase3 activity in AML-12 cells. (**I**) Cell viability of AML-12 cells. (**J**) Representative Western blots and quantification for apoptosis-related proteins in AML-12 cells. Data are presented as the mean ± SEM (*n* = 3 per group). * *p* < 0.05 compared with the Control group. ^#^ *p* < 0.05 compared with the PA group.

We continued to explore the effects of LNT on oxidative stress in AML12 cells. DHE staining indicated severe oxidative stress after 24 h of exposure to PA, which was prominently improved by LNT (Figure 4A). In addition, there were significantly higher levels of MDA and 4-HNE, as well as lower levels of SOD and GSH/GSSG in PA-induced AML12 cells compared with the Control group. These changes were prevented by LNT (Figure 4B–E). Furthermore, LNT significantly attenuated the decrease in the protein levels of anti-oxidative enzymes (SOD1, SOD2) and the increase in the protein levels of oxidative enzymes (NOX2, NOX4) after PA treatment in AML12 cells (Figure 4F).

To further prove the effects of LNT on apoptosis in vitro, flow cytometry showed that the percentage of apoptotic cells markedly increased in PA-induced AML12 cells, but significantly decreased under LNT treatment (Figure 4G). Consistently, the results of CCK8 revealed increased cell viability under the LNT treatment compared with the PA group (Figure 4I). Cleaved-Caspase3 protein levels and Caspase3 activity were significantly increased by palmitic acid addition, an effect that was also attenuated by LNT (Figure 4H). LNT attenuated the increase in pro-apoptotic Bax and the decrease in anti-apoptotic Bcl-2 after the addition of palmitic acid to AML12 cells, which may contribute to the anti-apoptotic effects of LNT on AML12 cells (Figure 4J). Collectively, these findings demonstrated that LNT may attenuate PA-induced oxidative stress and apoptosis in AML12 cells, which was consistent with the results obtained in NAFLD mice. 

### 2.5. PPARα Knockdown Abolished the Protective Effects of LNT on Lipid Deposition in PA-Induced AML12 Cells

**Figure 5:**
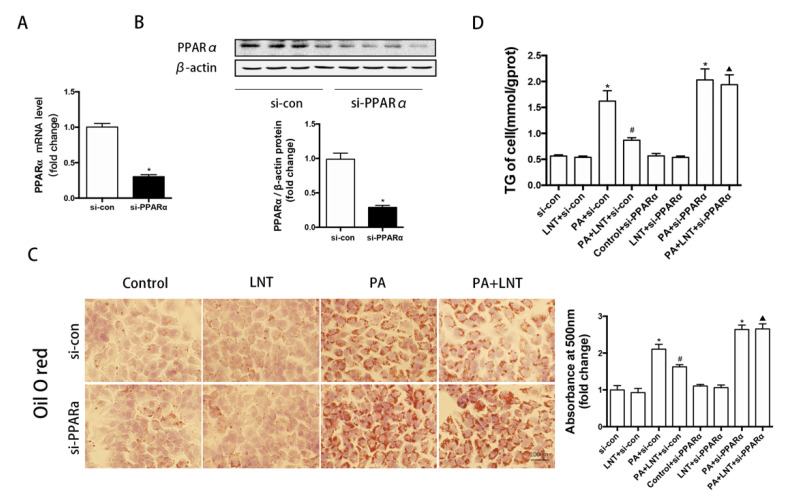
PPARα knockdown abolished the protective effects of LNT on lipid deposition in PA-induced AML12 cells. (**A**,**B**) PPARα mRNA and protein expression levels in AML-12 cells. (**C**,**D**) Oil red O staining and TG content in AML-12 cells. Data are presented as the mean ± SEM (*n* = 3 per group). * *p* < 0.05 compared with the si-con group. ^#^ *p* < 0.05 compared with the PA+si-con group. ^▲^ *p* < 0.05 compared with the PA+LNT + si-con group.

To investigate the roles of PPARα on the effects of LNT against PA-induced damage, a transfection approach using PPARα siRNA was carried out to knockdown PPARα. As expected, PPARα knockdown significantly decreased the expression levels of PPARα in both mRNA and protein in AML12 cells (Figure 5A,B). The decreased hepatocyte lipid droplets and triglyceride content seen under LNT treatment were prevented by pretreatment with PPARα siRNA in PA-induced AML12 cells (Figure 5C,D). This finding suggested that LNT exerted its ameliorating effect through PPARα.

### 2.6. PPARα Knockdown Prevented the Effects of LNT on Oxidative Stress and Apoptosis in PA-Induced AML12 Cells

**Figure 6:**
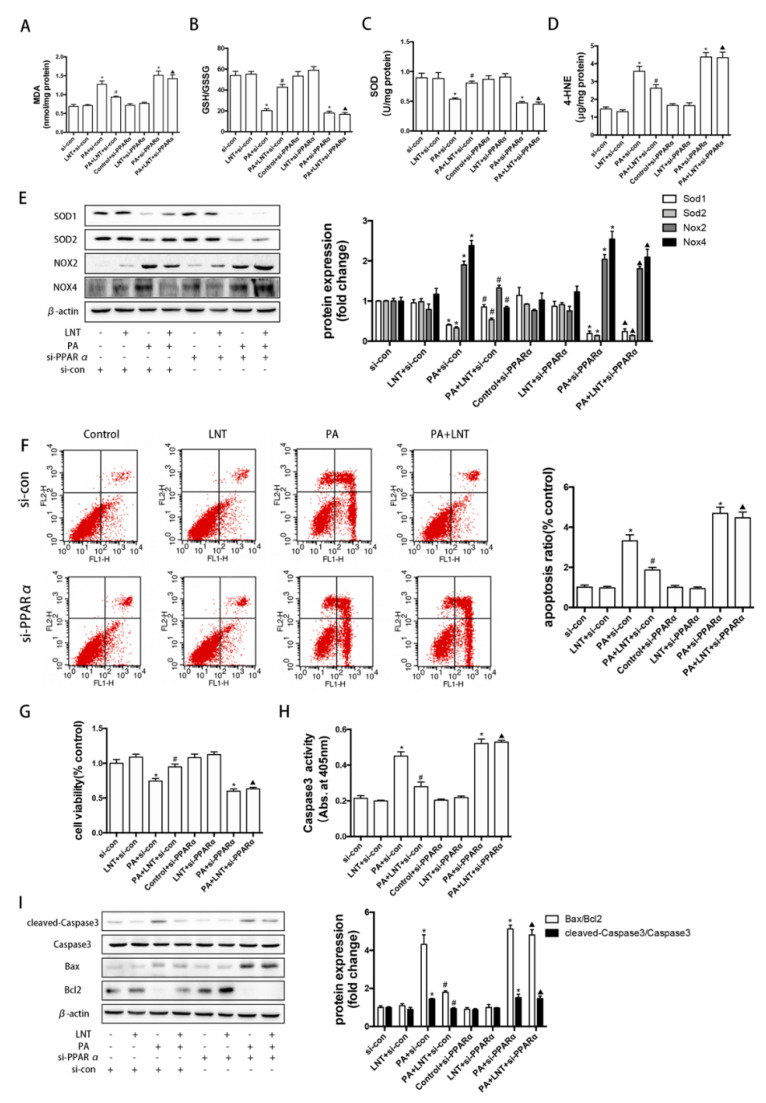
PPARα knockdown abolished the effects of LNT on oxidative stress and apoptosis in PA-induced AML12 cells. (**A**–**D**) MDA, GSH/GSSH, SOD, and 4-HNE levels in AML-12 cells. (**E**) Representative Western blots and quantification of SOD1, SOD2, NOX2, and NOX4 in AML-12 cells. (**F**) Representative annexin-V-FITC/PI staining and quantification of apoptosis by flow cytometry. (**G**) Cell viability and (**H**) Caspase3 activity in AML-12 cells. (**I**) Representative Western blots and quantification for cleaved Caspase3, apoptosis-related proteins in AML-12 cells. Data are presented as the mean ± SEM (*n* = 3 per group). * *p* <0.05 compared with the si-con group. ^#^ *p* < 0.05 compared with the PA+si-con group. ^▲^ *p* < 0.05 compared with the PA+LNT + si-con group.

We further investigated the roles of PPARα on the effects of LNT against PA-induced oxidative stress and apoptosis. LNT alleviated PA-mediated increased MDA and 4-HNE levels and decreased GSH/GSSG and SOD levels in AML12 cells. However, PPARα siRNA abolished these effects of LNT (Figure 6A–D). Similarly, as shown in (Figure 6E), PPARα siRNA blocked the LNT-induced upregulation of SOD1 and SOD2 protein expression levels and the downregulation of NOX2 and NOX4 protein expression levels in PA-treated AML12 cells. Flow cytometry revealed a significant decrease in PA-induced apoptosis after treatment with LNT compared with PA-only addition; these protective effects of LNT were abolished by PPARα siRNA (Figure 6F). PPARα knockdown also attenuated the LNT-induced increase in cell viability in PA-treated AML12 cells (Figure 6G). Furthermore, LNT alleviated the PA-mediated elevation of cellular Caspase3 activity, cleaved-Caspase3 protein levels, and the Bax/Bcl-2 ratio, which were prevented by PPARα knockdown (Figure 6H,I). These findings indicated that LNT attenuated PA-induced oxidative stress and apoptosis via the activation of PPARα in vitro. 

## 3. Discussion

In the present study, we tested the effects of LNT on a well-established NAFLD mouse model, which was induced by continuous HFD feeding. We found that LNT attenuated the detrimental effects associated with NAFLD. Specifically, LNT decreased liver cholesterol and triglycerides, improved hepatic steatosis, and attenuated oxidative stress and apoptosis. LNT also upregulated the expression of the PPARα signal pathway, which was typically suppressed in NAFLD. Together, our results suggested that LNT protected against the development and progression of NAFLD at least partially through the PPARα pathway in this model.

β-glucans are heterogeneous non-starch polysaccharides, which form the structural compounds of the cell wall of certain microorganisms, including algae and yeast, and certain protists, including mushrooms and grains, such as wheat and oats [17]. β-glucan, the main component of LNT, plays a key role in LNT function, including immunomodulation, antiviral activity, antitumor activity, antioxidation and anti-inflammation [18,19,20,31]. β-glucans have been used to reduce blood cholesterol levels since the 1960s [32]. On the basis of the extensive evidence showing an inverse association between β-glucan intake and LDL cholesterol, several countries have currently approved the health claims of oat β-glucan for its LDL-C lowering effects and its ability to reduce CVD risk [17,33,34]. In addition, agaricus β-glucan was proved to have anti-hyperglycemic, anti-triglyceride, and anti-atherosclerosis effects in diabetic rats [35]. In this study, LNT significantly reduced TC and triglycerides both in vivo and in vitro, which was consistent with a previous study reporting that shiitake (*Lentinus edodes*) and maitake had a cholesterol-lowering effect in spontaneously hypertensive rats [21]. 

Oxidative stress, which is produced by large amounts of ROS, has increasingly emerged as the pivotal factor in the development and progression of NAFLD through the induction of lipid peroxidation and the promotion of lipid accumulation, insulin resistance, and inflammation [12]. ROS, which are primarily superoxide anions, hydrogen peroxide, and free radicals, are considered to be closely associated with lipid peroxidation, and irretrievable protein and DNA degeneration. The resulting extremely reactive aldehyde components, such as 4-HNE and MDA, can be used as representative biomarkers of lipid peroxidation [10]. NADPH oxidases (NOXs) are major producers of ROS. Redundant ROS are generally eliminated by a series of enzymes, primarily including superoxide dismutase (SOD), glutathione peroxidase (GPx), and catalase (CAT), whereas the nonenzymatic molecules include glutathione (GSH), beta-carotene, and tocopherol [12,36]. When the antioxidant system is vulnerable, the expression of activated ROS will be enhanced. In our result, LNT significantly reduced the activity of ROS, MDA, 4-HNE, NOX2, and NOX4, while the ratio of GSH/GSSH and the content of SOD1 and SOD2 were significantly elevated both in vivo and in vitro. However, after co-treatment with PPARα siRNA, the ratio of GSH/GSSH and the content of SOD1 and SOD2 decreased, and the activity of ROS, MDA, NOX2, and NOX4 increased, indicating that LNT restored redox balance in NAFLD, at least partly through the PPARα pathway by enhancing cellular antioxidant activity and improving the ability of cells to scavenge ROS. Zi. et al. reported that benzo(a)pyrene induced oxidative damage in human immortalized keratinocytes (HaCaT cells) was notably rescued by LNT treatment, consistent with our results [37].

Apoptosis plays an important role in the progression of NAFLD. Bcl-2 protein, which is mainly located at the outer part of the mitochondrial membrane, may play a key role in regulation of the mitochondrial–apoptosis system and is a member of the regulatory proteins in the complex apoptosis pathway. The Bax gene plays a key role in the process of apoptosis. The ratio of Bax to Bcl-2 is called the “apoptosis switch”, as cell apoptosis occurs when the Bax protein is dominant. Caspase3 is the most critical protease responsible for mediating and executing death instructions and is a key effector of apoptosis in hepatocytes [38]. A previous study pointed out that Caspase3 knockout in mice was associated with decreased levels of apoptosis in NASH induced by a methionine- and choline-deficient (MCD) diet [39]. In our results, LNT significantly reduced the ratio of Bax/Bcl2 protein and Caspase3 activity both in vivo and in vitro. After co-treatment with PPARα siRNA, the ratio of Bax/Bcl2 protein and Caspase3 activity increased, indicating that LNT acted at least partly through the PPARα pathway to reduce apoptosis. Former studies pointed out that LNT afforded significant protection against paclitaxel-induced apoptosis in mouse bone marrow cells [40]. In addition, Zhang et al. pointed out that the level of apoptosis significantly decreased in LNT-treated pancreatic beta-cells compared with STZ-induced cells [41]. 

PPARα has been widely accepted as a transcriptional switch for various genes involved in liver FA uptake and oxidation. PPARα activation protected against HFD-induced hepatocellular injury and liver inflammation and improved insulin sensitivity [25]. Similarly, PPARα-deficiency in mice promoted HFD-induced hepatic TG and macrophage infiltration, and elevated plasma levels of ALT and AST [28]. In addition, hepatocyte-specific PPARα deletion in mice showed a marked increase in hepatic steatosis, increased plasma FFA, and impaired ketone bodies in response to two weeks of HFD feeding [42]. In this study, we found that LNT upregulated the expression of PPARα and its target gene CPT1α, which is involved in lipid metabolism in the liver of NAFLD mice, as well as in AML12 cells in vitro. In addition, after siRNA-PPARα transfection, LNT had little meliorative effect on PA-induced cells with a low expression of PPARα, confirming that PPARα activation plays a key role in LNT attenuation of NAFLD.

Carnitine palmitoyl transferase 1α (CPT1α) is a subtype of the carnitine palmitoyl transferase (CPT) enzyme family, which is mainly expressed in the liver. CPT1α is located in the outer membrane of the mitochondria and is one of the rate-limited enzymes of lipid metabolism. By converting acyl CoA to acyl carnitine, CPT1α is in charge of the transportation of fatty acids into the mitochondria for further oxidation [43]. It is well known that de novo lipogenesis and β-oxidation of fatty acids are two key roles in lipid metabolism. PPARα and CPT1α are two key enzymes in the β-oxidation of fatty acids [44]. Importantly, CPT1α is a target gene of PPARα, and the mechanism of PPARα for transporting fatty acids into hepatocyte mitochondria could be associated with the increasing effect of PPARα on CPT1α in the liver, which also reveals the consistency of expression between PPARα and CPT1α [45]. In this study, LNT significantly increased the mRNA and protein expression levels of PPARα and CPT1α and decreased both serum and liver TC and triglyceride levels. These results were consistent with those of previous studies, which pointed out that CPT1α upregulation leads to a reduction in triglycerides. In addition, CPT1α activation was related to the decrease in TC and LDL-C, as the long-chain fatty acids can be converted into triglycerides via the transshipment of the CPT enzyme system. Overexpression of CPT1α attenuated NAFLD by activating β-oxidation [46], whereas HFD-fed CPT1α heterozygous knockout mice had more severe hepatic lipid accumulation [47]. This study suggested that the PPARα/CPT1α pathway plays an important role in LNT’s attenuation of NAFLD. 

## 4. Materials and Methods 

### 4.1. Animals

The animal experiments were consistent with ARRIVE and NIH guidelines for animal welfare [48], and were conducted with the approval of the Animal Research Committee of Tongji Medical College, Huazhong University of Science and Technology, Wuhan, China. Male C57BL/6J mice (5–6 weeks old) were purchased from Beijing Vital River Laboratory Animal Technology Co., Beijing, China. Mice were given free access to food and water and housed with a 12-h light/dark cycle. After acclimatization for 1 week, mice were randomly fed with either a standard laboratory chow diet or a 60% high-fat diet (diet#D12492) for 6 weeks. The mice were then randomly divided into 4 groups (8 mice/group): (1) Control, fed with a chow diet; (2) LNT, fed with a chow diet and orally treated with LNT at 6 mg/kg/d; (3) HFD, fed with a 60% high-fat diet; (4) HFD + LNT, fed with a 60% high-fat diet and orally treated with LNT at 6 mg/kg/d. The oral concentration was calculated as described previously [49]. Weight gain, blood glucose, and serum lipids were measured every 3 weeks and all the data were collected blindly. All animals were sacrificed on the 21st week after beginning the diet. For tissue sampling, mice were fasted overnight and anesthetized with pentobarbital (50 mg/kg bodyweight). Blood was collected from the retro-orbital vein. Parts of the liver were obtained for histological analysis; RNA extraction, and protein analysis; frozen with liquid nitrogen; and stored at −80 °C. The flow chart of the animal experiment is shown in Supplementary Materials Figure S1.

### 4.2. Cell Culture and Treatments

Mouse alpha liver cells (AML12), an immortalized normal mouse hepatocyte cell line, were cultured in Dulbecco’s modified Eagle’s medium (DMEM) with 10% FBS in an atmosphere of 95% air and 5% CO_2_ at 37 °C. After synchronization, cells were treated with or without LNT (5 μg/mL) for 0.5 h. Next, the cells were incubated with 1.0% bovine serum albumin (BSA) alone or in combination with 0.4 mM free fatty acid (FFA) containing 1.0% acid-free BSA for 24 h. The concentration and intervention methods were obtained according to previous descriptions [31] and a prior experiment (Supplementary Materials Figure S2).

The silencing of peroxisome proliferator-activated receptor α (PPARα) was performed by small interfering RNAs (siRNAs), synthesized by Ribobio (Guangzhou, China). The siRNA transfection was conducted with Lipofectamine 2000 (Invitrogen, Waltham, MA, USA) according to the manufacturer’s protocol. After 24 h of transfection, cells were cultured with 5 μg /mL LNT and 0.2 mM palmitate, as shown above, for another 24 h. Western blot was used to determine the siRNA knockdown efficiency 36 h after transfection. Experiments were subsequently performed only if the silencing effect was more than 70%.

### 4.3. Biochemical Parameters

Total cholesterol (TC), low-density lipoprotein cholesterol (LDL-C), high-density lipoprotein cholesterol (HDL-C), and triglyceride (TG) in serum, liver, or AML12 cells were measured by ELISA according to the manufacturer’s protocol (Nanjing Jiancheng Bioengineering Institute, Nanjing, China). MDA, GSH, SOD, ALT, AST, ALP, BA, TBIL, TNF-α, IL-6, and IL-10 levels and Caspase3 enzyme activity in serum, liver tissue, or cell homogenate were also measured by ELISA according to the manufacturer’s protocol (Beyotime, Shanghai, China). Moreover, 4-HNE levels in liver tissue and cells were measured by ELISA according to the manufacturer’s protocol (Abcam, Shanghai, China).

### 4.4. Western Blot Analysis

Western blot analysis was performed as previously described [50]. Briefly, equal amounts of protein from liver tissue or AML12 cells were separated by electrophoresis on 10% SDS-PAGE gels. The resolved proteins were electrophoretically transferred to polyvinylidene difluoride membranes using a transfer buffer containing 192 mM glycine, 20% (*v*/*v*) methanol, and 0.02% SDS. The membranes were incubated with 5% nonfat dry milk in TBST for 2 h, then incubated overnight at 4 °C with the indicated primary antibodies. The proteins were visualized by enhanced chemiluminescence (ECL) and quantified by Image J software. The antibodies used in the study are listed in Supplementary Materials Table S1.

### 4.5. Histological Analysis

Liver tissues, fixed in 10% formalin, were embedded in paraffin, sectioned into 4 μm slices, and stained with H&E as previously described [51]. Frozen liver sections (8 μm) and cells in 6-well plates were stained with oil red O and DHE (Sigma-Aldrich, MO, USA) to assess lipid accumulation and ROS accumulation based on the requirements of the manufacturer’s protocol. All the sections were visualized with a microscope and quantified by Image-Pro Plus Version 6.0.

### 4.6. Flow Cytometry Analysis

AML12 cells were cultivated with Annexin V-FITC/PI (BD Biosciences, Sparks, MD, USA) to assess cell apoptosis. After cultivation, cells were analyzed with a FACStar Plus flow cytometer (BD Biosciences, Sparks, MD, USA), as described previously [52].

### 4.7. Quantitative RT-PCR

RNA from cells and animal tissues was isolated by TRIzol, and then reserved and transcribed into cDNA by means of the First-Strand cDNA Synthesis Kit (Vazyme Biotech, Nanjing, China). Real-time PCR was carried out with the SYBR rapid quantitative PCR Kit (Vazyme Biotech, Nanjing, China). All processes were conducted in accordance with the manufacturer’s protocol. The primers that were used in the study are listed in Supplementary Materials Table S2.

### 4.8. Statistics

All data are displayed as means ± SEM, and discrepancies among groups were determined by Student’s *t*-test or ANOVA after numerous comparisons with Tukey’s test. All these statistical tests were conducted with SPSS (v18.0), and *p* < 0.05 was regarded as statistically significant in all cases.

## 5. Conclusions

In conclusion, we demonstrated that the LNT improved the lipid accumulation, oxidative stress, and apoptosis of NAFLD, at least partly though the PPARα pathway. LNT may be a potential drug target for NAFLD.

## Data Availability

All data is contained within the article and Supplementary Materials.

## Supplementary Materials

The following are available online at https://www.mdpi.com/article/10.3390/metabo12010055/s1, Figure S1. Flow chart of the animal experiment, Figure S2. Effects of different concentrations of LNT in PA-induced AML12, Figure S3. Effects of LNT on liver function in HFD-induced mice, Figure S4. Effects of LNT on inflammatory cytokines in HFD-induced mice, Figure S5. Effects of LNT on related protein in PA-induced AML12 cells, Table S1. List of antibodies, Table S2. List of primer sequences.

## Abbreviations

ACAT, acyl-coenzyme A: cholesterol acyltransferase; APOB, apolipoprotein B; BA, bile acid; ALT, alanine aminotransferase; ALP, alkaline phosphatase; AML12, alpha mouse liver 12; AMPKα, adenosine 5’-monophosphate (AMP)-activated protein kinase-α; AST, aspartate transaminase; CD36, cluster of differentiation 36; CPT1α, carnitine palmityl transferase 1α; CHREBP, carbohydrate response element binding protein; FAS, fatty acid synthetase; FXR, farnesoid X receptor; HDL-C, high-density lipoprotein cholesterol; HFD, high-fat diet; IL-6, interleukin-6; IL-10, interleukin-10; LDL-C, low-density lipoprotein cholesterol; LNT, lentinan; LXR, liver X receptor; MTTP, microsomal triglyceride transfer protein; PA, palmitic acid; PPARα, peroxisome proliferator-activated receptor α; NAFLD, nonalcoholic fatty liver disease; NASH, nonalcoholic steatohepatitis; NOX2, NADPH oxidase 2; NOX4, NADPH oxidase 4; ROS, reactive oxygen species; RXRα, retinoic acid X receptor α; SIRT1, silence information regulator 1; SOD1, superoxide dismutase 1; SOD2, superoxide dismutase 2; SREBP, sterol regulatory element binding protein; STZ, streptozotocin; TBIL, total bilirubin; TC, total cholesterol; TG, triglycerides; TNF-α, tumor necrosis factor; 4-HNE, 4-hydroxy-2-nonenal.
