# Supplementary material for: Lentinan Protects against Nonalcoholic Fatty Liver Disease by Reducing Oxidative Stress and Apoptosis via the PPARα Pathway
Source: Metabolites. 2022 Jan 10;12(1):55. doi: 10.3390/metabo12010055 (PMC8780611; doi:10.3390/metabo12010055)
Supplement: Supplementary file 1 [file metabolites-12-00055-s001.zip › metabolites-1538015-SI.pdf]

Lentinan Protects against Nonalcoholic Fatty Liver Disease by Reducing Oxidative Stress and Apoptosis via the PPARα Pathway

Tingyi Du [1,2], Qin Fang [1], Zhihao Zhang [1], Chuanmeng Zhu [1], Renfan Xu [3], Guangzhi Chen [1,*] and Yan Wang [1,*]

[1] Division of Cardiology, Department of Internal Medicine, Tongji Hospital, Tongji Medical College, Huazhong University of Science and Technology, Wuhan 430030, China
[2] Department of Radiology, Zhongnan Hospital of Wuhan University, Wuhan 430071, China
[3] Department of Medical Ultrasound, Tongji Hospital, Tongji Medical College, Huazhong University of Science and Technology, Wuhan 430030, China
* Correspondence: chengz2003@163.com (G.C.); newswangyan@tjh.tjmu.edu.cn (Y.W.); Tel./Fax: +86-27-6937-8422 (G.C. & Y.W.)

Online supplemental data

SUPPLEMENTAL TABLES

Table S1. List of antibodies.

Antibody	Company	Catalog number
AMPKα	Abclonal	A12718
p-AMPKα	Abclonal	AP0116
BCL2	Proteintech	No. 66799-1-Ig
BAX	Proteintech	No. 60267-1-Ig
Caspase3	Abclonal	A2156
cleaved-Caspase3	Abclonal	A19654
CPT1α	Abclonal	A5307
PPARα	Abclonal	A6697
SOD1	Abclonal	A0274
SOD2	Abclonal	A1340
NOX2	Abclonal	A19701
NOX4	Abclonal	A11274
RXRα	Abclonal	A19105

Table S2. List of *p*rimer sequences.

Gene	Sequences
Pparα-F:	5′ GGCAATGGCTTTATCACACG 3′
Pparα-R:	5′ TGTCATCCAGTTCTAAGGCATTG 3′
Chrebp-F:	5′ CTCAACTCCATACAACCCTCG 3′
Chrebp-R:	5′ CGTCGGTTCTCCATCTTGTT 3′
Srebp1-F:	5′ GTTTCCGGGGAACTTTTCCT 3
Srebp1-R:	5′ TGAGCTGGAGCATGTCTTCG 3′
Cd36-F:	5′ AGCAGTGATTTGACTTGTGGC 3′
Cd36-R:	5′ AAGGCTAGGAAACCATCCACC 3′
Cpt1α-F:	5′ GAGAATGCCAGGAGGTCATAGAG 3′
Cpt1α-R:	5′ AGTCTCTGTCCTCCCCTCTCG 3′
Pparγ-F:	5′ ACCACTCGCATTCCTTTGACA 3′
Pparγ-R:	5′ TCGCACTTTGGTATTCTTGGAG 3′
Acat-F:	5′ CATCCAAGGGGGTGAAGGAC 3′
Acat -R:	5′ GGTGTTGCTCCTCTGCTCAT 3′
Apob-F:	5′ TGCAGTCAACAGCAAGAGCA 3′
Apob -R:	5′ CCTCCATCCTGAGTTGGACA 3′
Mttp-F:	5′ AGAGCATCTTCCAGGGCAAA 3′
Mttp -R:	5′ TATGCCCACTGGCTCGTTTT 3′
Lxr-F:	5′ CAATGCCTGATGTTTCTCCTGAT 3′
Lxr -R:	5′ GCTCTGCTGACTCCAACCCTAT 3′
Fxr-F:	5′ ATTTACAGGCTACGGACGAGTTT 3′
Fxr-R:	5′ GTAGAAGCCCAGGTTGGAATAGTA 3′
Rxrα-F:	5′ CACATTGGGCTTCGGGACT 3′
Rxrα-R:	5′ AGCGGTCCCCACAGATAGC 3′
Sirt1-F:	5′ AGCGGCTTGAGGGTAATCAA 3′
Sirt1-R:	5′ TTCGGGCCTCTCCGTATCAT 3′
Gapdh-F:	5′ CAAAATGGTGAAGGTCGGTGTG 3′
Gapdh -R:	5′ TGATGTTAGTGGGGTCTCGCTC 3′
Tnfα-F:	5′ CAGGCGGTGCCTATGTCTC 3′
Tnfα-R:	5′ CGATCACCCCGAAGTTCAGTAG 3′
Il-6-F:	5′ TCTATACCACTTCACAAGTCGGA 3′
Il-6-R:	5′ GAATTGCCATTGCACAACTCTTT 3′
Il-10-F:	5′ CAGCCGGGAAGACAATAACT 3′
Il-10-R:	5′ GCATTAAGGAGTCGGTTAGCA 3′
Fas-F:	5′ GGAGGTGG TGATAGCCGGTAT 3′
Fas-R:	5′ TGGGTAATCCATAGAGCCCAG 3′

SUPPLEMENTAL FIGURES

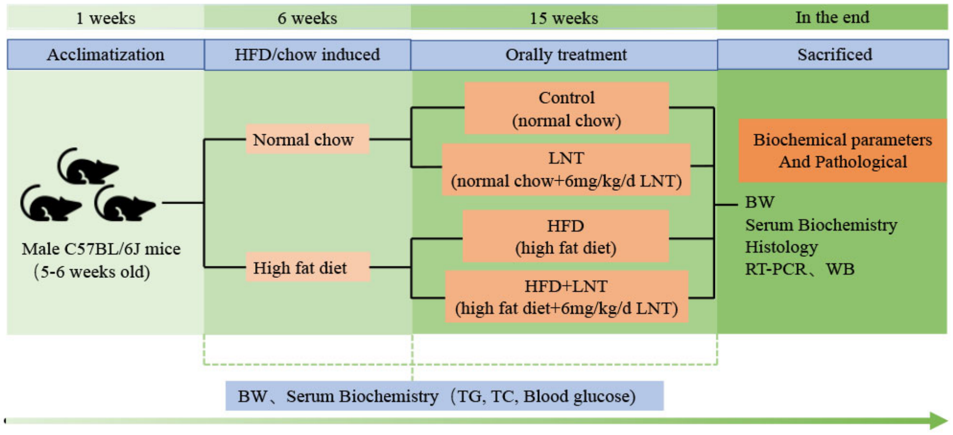

Figure S1. Flow chart of the animal experiment.

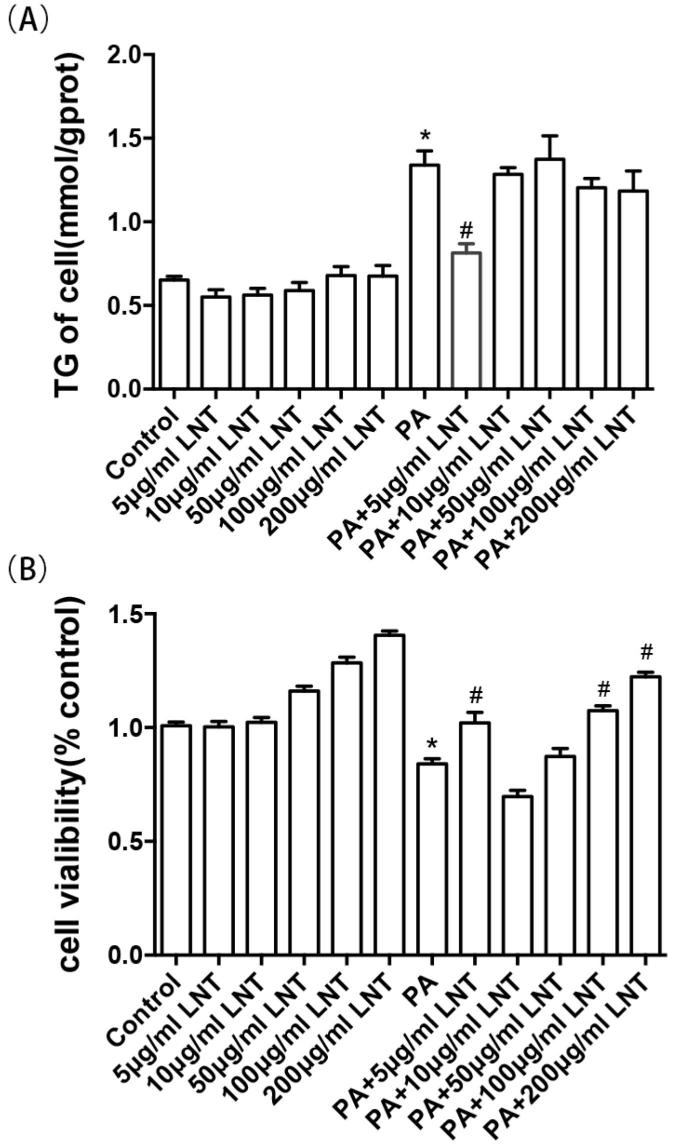

Figure S2. Effects of different concentrations of LNT in PA-induced AML12. (**A**) TG content and (**B**) cell viability in AML-12 cells by PA and different concentrations of LNT treatment. Data are presented as the mean ± SEM (n = 3 per group). $^{*}p < 0.05$ compared with the Control group. $^{\#}p < 0.05$ compared with the PA group.

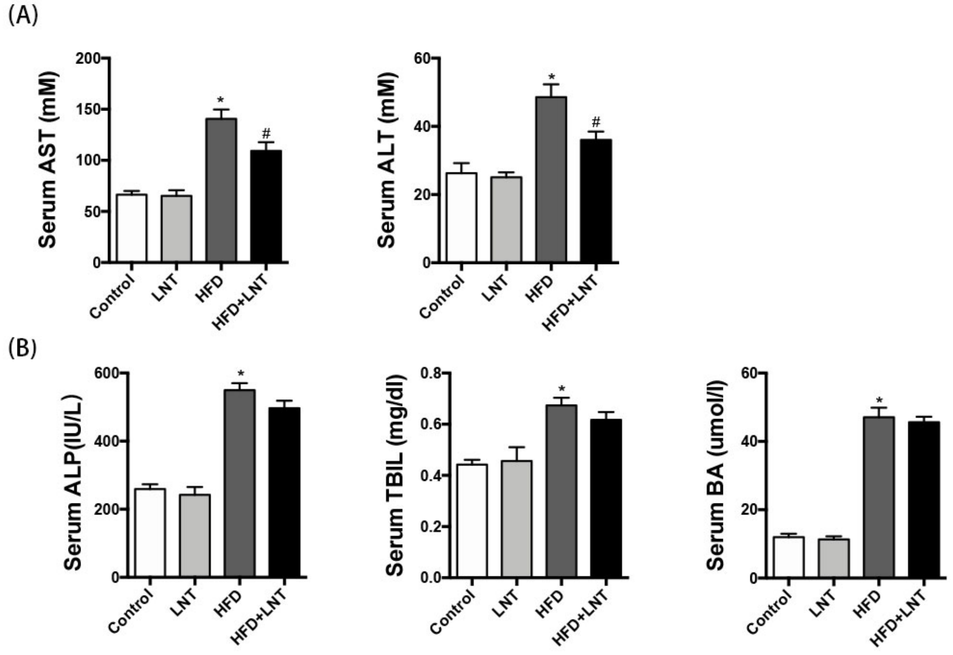

Figure S3. Effects of LNT on liver function in HFD-induced mice. (**A**) AST and ALT, and (**B**) ALP, TBIL, and BA levels in serum. Data are presented as the mean ± SEM (n = 8 per group). $^{*}p < 0.05$ compared with the Control group. $^{\#}p < 0.05$ compared with the HFD group.

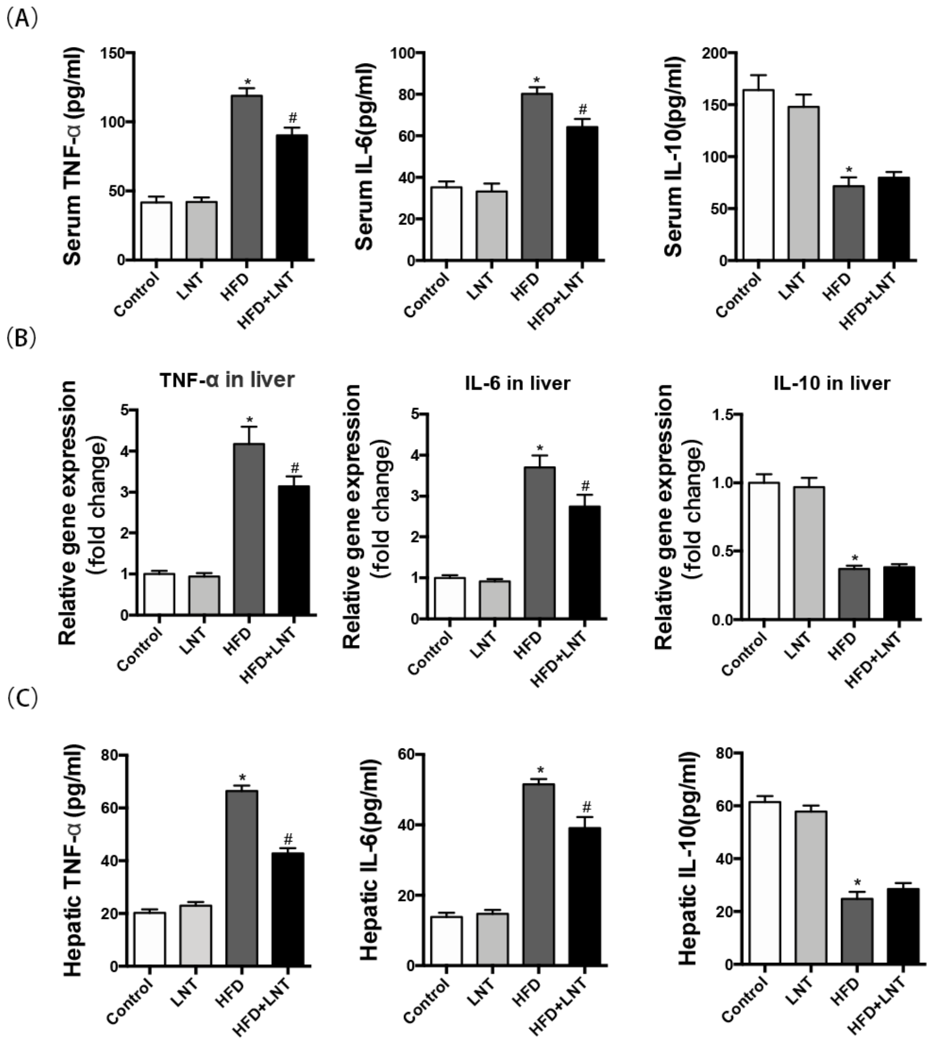

Figure S4. Effects of LNT on inflammatory cytokines in HFD-induced mice. (**A**) Serum levels of TNF-α, IL-6, and IL-10 in four groups of mice. (**B**) Hepatic mRNA and (**C**) protein levels of TNF-α, IL-6, and IL-10. Data are presented as the mean ± SEM (n = 8 per group). * $p < 0.05$ compared with the Control group. $^{\#}$ $p < 0.05$ compared with the HFD group.

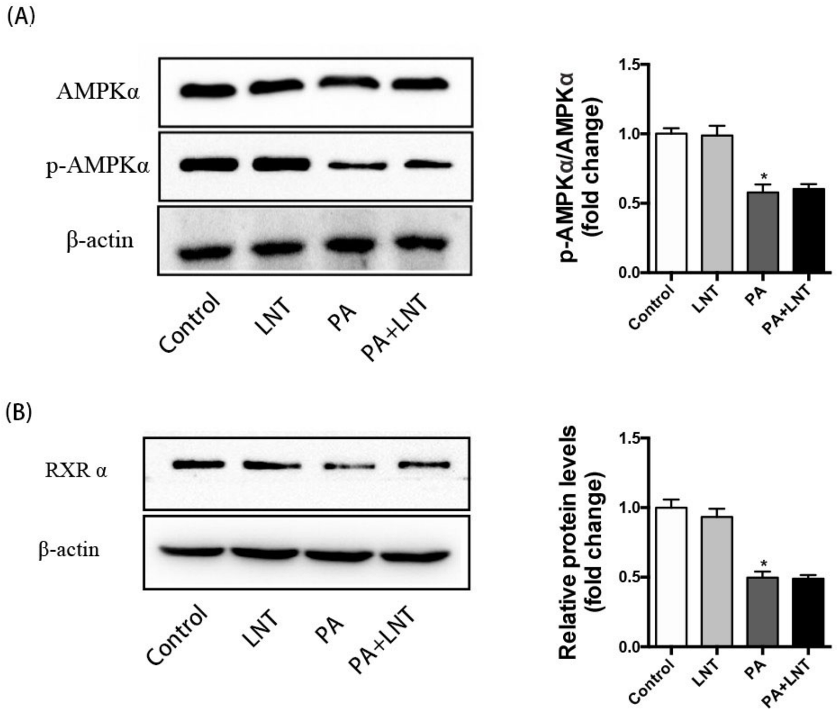

Figure S5. Effects of LNT on related genes in PA-induced AML12 cells. Representative Western blots and quantification of (**A**) AMPKα, p-AMPKα and (**B**) RXRα in AML-12 cells. Data are presented as the mean ± SEM (n = 3 per group). $^{*}p < 0.05$ compared with the Control group. $^{\#}p < 0.05$ compared with the PA group.
